# Influence of Resin Cement Thickness and Elastic Modulus on the Stress Distribution of Zirconium Dioxide Inlay-Bridge: 3D Finite Element Analysis

**DOI:** 10.3390/polym13223863

**Published:** 2021-11-09

**Authors:** Joseph Assaf, Louis Hardan, Cynthia Kassis, Rim Bourgi, Walter Devoto, Elie Amm, Carol Moussa, Jacek Sawicki, Monika Lukomska-Szymanska

**Affiliations:** 1 Department of Restorative Dentistry, School of Dentistry, Saint-Joseph University, Beirut 1107 2180, Lebanon; joseph.assaf@net.usj.edu.lb (J.A.); cynthia.kassis@usj.edu.lb (C.K.); rim.bourgi@net.usj.edu.lb (R.B.); carol.moussa@net.usj.edu.lb (C.M.); 2 Independent Researcher, 16030 Sestri Levante, Italy; walter@walterdevoto.com; 3 Department of Orthodontics, School of Dental Medicine, Saint Joseph University, Beirut 1107 2180, Lebanon; elie.el-amm@usj.edu.lb; 4 Institute of Materials Science and Engineering, Lodz University of Technology, 90-924 Lodz, Poland; jacek.sawicki@p.lodz.pl; 5 Department of General Dentistry, Medical University of Lodz, 92-213 Lodz, Poland

**Keywords:** adhesive prosthesis, cement spacer, elastic modulus, finite element analysis, inlay bridge, resin cement

## Abstract

The mechanical properties and the thickness of the resin cement agents used for bonding inlay bridges can modify the clinical performance of the restoration such as debonding or prosthetic materials fracture. Thus, the aim of this study was to evaluate the stress distribution and the maximum strain generated by resin cements with different elastic moduli and thicknesses used to cement resin-bonded fixed partial denture (RBFPD). A three-dimensional (3D) finite element analysis (FEA) was used, and a 3D model was created based on a Cone-Beam Computed Tomography system (CBCT). The model was analyzed by the Ansys software. The model fixation occurred at the root of the abutment teeth and an axial load of 300 N was applied on the occlusal surface of the pontic. The highest stress value was observed for the Variolink 0.4 group (1.76 × 10^6^ Pa), while the lowest was noted for the Panavia 0.2 group (1.07 × 10^6^ Pa). Furthermore, the highest total deformation value was found for the Variolink 0.2 group (3.36 × 10^−4^ m), while the lowest was observed for the Panavia 0.4 group (2.33 × 10^−4^ m). By means of this FEA, 0.2 mm layer Panavia F2.0 seemed to exhibit a more favorable stress distribution when used for cementation of posterior zirconium-dioxide-based RBFPD. However, both studied materials possessed clinically acceptable properties.

## 1. Introduction

The ideal treatment option is to replace a missing tooth with a dental implant [1]. However, this option can be expensive, and many complications may occur due to medical (i.e., uncontrolled diabetes, several cancer therapies) and surgical conditions (i.e., insufficient bone volume) [2]. In these cases, an alternative treatment would be the conventional three-unit porcelain fused to metal (PFM) bridge. Nevertheless, when preparing a tooth for a full-coverage crown, approximately 63% to 73% of its coronal structure is inevitably lost [3]. This loss may lead to irreversible pulpitis or pulpal necrosis in 15.6% of vital crowned teeth [1].

Therefore, RBFPD seems to be a more conservative, economic alternative to restore a missing tooth in both anterior and posterior regions [4]. Unaesthetic metal frameworks have been replaced by composite resin-based and all-ceramic RBFPDs. Different materials (zirconium dioxide, lithium disilicate, chrome/cobalt alloy) have been used for fabrication of RBFPDs [5,6]. Zirconium dioxide provides mechanical properties of alloy restoration and esthetic properties of lithium disilicate ceramics.

Many studies have focused on the elastic modulus of the cement, but few have included the thickness of the resin cement, knowing that ‘de-bonding’ is the major cause of failure of RBFPD [7,8]. To overcome these failures, an understanding of the tensile stress distribution in the tooth/cement/prosthesis complex is crucial [8]. Hence, it is important to know how the elastic modulus properties of the resin cement can modify the stress generated at the cement/bridge interface. Therefore, the selection of a cement with an optimal elastic modulus and thickness could improve the stress distribution during masticatory loads and consequently prolong the survival of RBFPD.

The comprehensive evaluation of the complex formed by the restorative material and teeth structure presents methodological difficulties. FEA was introduced to overcome these limitations [8,9]. It is a well-established, mathematical method that studies the biomechanical behavior of dental structures and materials (i.e., strain and stress distribution) in a controlled condition, often difficult to reproduce in vitro. Consequently, periodontal ligaments and medullar and cortical bone can be simulated, which is impossible in a conventional in vitro study [10,11].

The aim of this study was to evaluate the stress distribution and the maximum strain generated by resin cements with different elastic moduli and thicknesses used to cement RBFPD. Hence, the null hypothesis was: (1) the elastic modulus of the cement resin does not influence the stress and strain exerted on the inlay bridge, and (2) the resin cement thickness does not have any effect on the stress distribution among the bridge.

## 2. Materials and Methods

### 2.1. Generation of the Geometric Models

**Figure 1:**
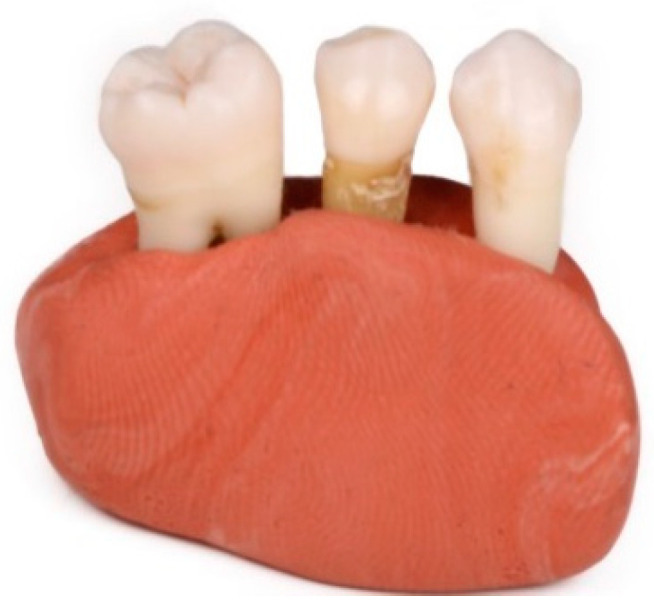
Extracted teeth placed in a silicone socket.

The 3D model was created based on a CBCT system (CBCT, CS 9600-Carestream Dental, Atlanta, GA, USA) of 3 extracted teeth (#44, #45, #46) placed in a silicone baseplate (Figure 1). The CBCT images were obtained from Trad Radiology Center (Kousba, Koura, Lebanon) and imported to the segmentation software Synopsys Simpleware Scan IP (M-2017.06 SP1-Student Edition, Mountain View, CA, USA). The body was manually segmented to remove any scan-related uncertainties. Then, the model was polished using a ratio of µ = 1.2. It is a ratio that reduces the original volume of the model in order to obtain a smooth model with no irregularities.

**Figure 2:**
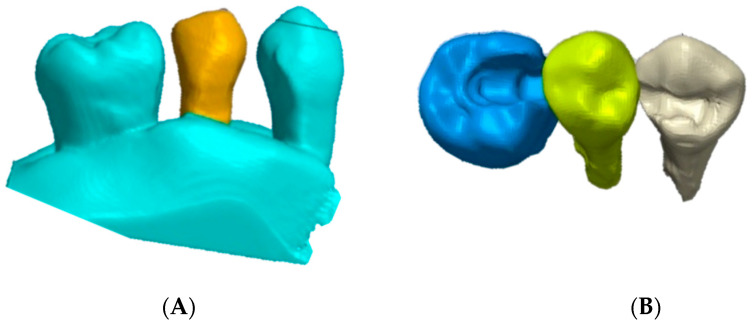
Digital cavity preparations: (**A**) frontal view; (**B**) occlusal view.

Before extraction of the second premolar from the digital model, the inlay cavity preparation was performed (6–10-degree axial walls and rounded internal angles). The premolar retainer was 2 mm in length, 2 mm in height, and 5 mm in width, while the molar retainer was 3 mm in length, 2 mm in height, and 8 mm in width (Figure 2).

**Figure 3:**
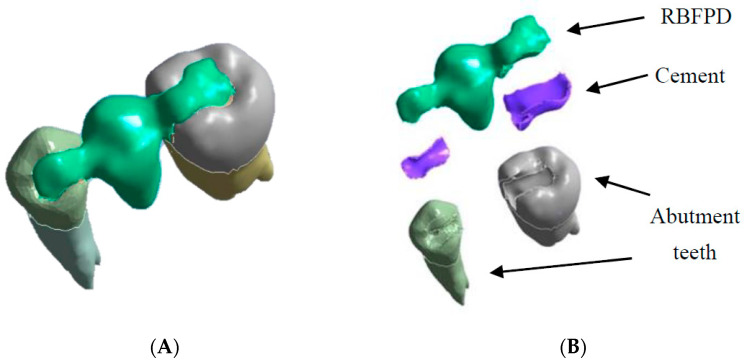
Geometric model of RBFPD used for numerical analysis: (**A**) bridge in situ; (**B**) elements presented separately.

Next, an inlay bridge was designed using the Ansys software (Ansys Workbench 2021 R1 Student Edition, Houston, TX, USA) of finished elements. Afterwards, the bridge was designed by adding two-dimensional (2D) pixels on every slice to obtain a 3D model, which was then polished twice using a ratio of µ = 1.2 (Figure 3).

**Figure 4:**
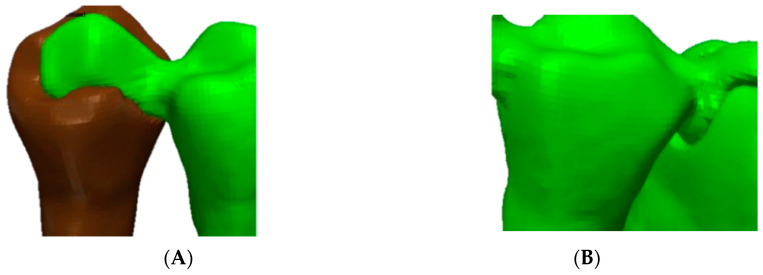
Connectors used in this study: (**A**) mesial connector; (**B**) distal connector.

The connectors used in this study had a circular shape with an area of 16 mm^2^ (Figure 4).

The physical properties of the materials used in the numerical model are shown in Table 1. Materials were modeled as isotropic entities. The adhesive bonding layer between the zirconium dioxide and cement was not included in the FEA, since the interface between all parts of the geometrical model is considered an ideal combined interface.

### 2.2. Study Design

Two cement resins with different elastic moduli and in two thicknesses were used. Therefore, four study groups were evaluated (Table 2).

### 2.3. Finite Element Analysis (FEA)

**Figure 5:**
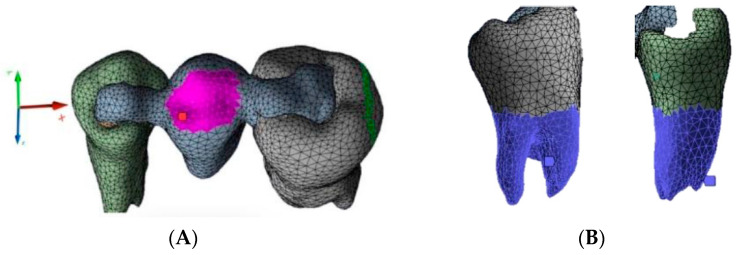
Discrete model and boundary conditions of the analyzed model: (**A**) loads (the purple area); (**B**) fixed support (the blue area).

The FEA method consists of dividing a structure (i.e., prosthesis, tooth) into a set of sub-domains called finished elements or mesh, bound together by knots (Figure 5). The discrete model was created using ANSYS Workbench software (Ansys Inc., Canonsburg, PA, USA). The generated grid consisted of adequately parameterized finite elements, enabling favorable and precise simulation results. The defined mesh subzones, combined with the earlier division of the geometry, enabled the optimization of the grid. Mesh convergence analysis was carried out by incrementally increasing the number of elements and verifying the estimations to ensure the convergence of the numerical solution. Finally, mesh convergence tests were performed, resulting in a total number of 9680 tetrahedral elements.

The load was applied using the force option. An occlusal load of 300 N was applied on the central surface of the pontic, as shown in Figure 5A. The model was restrained on the tooth root along the circumference using the ‘fixed support’ option (Figure 5B). A ‘bonded’ contact was used at the junction of all bodies. The 300 N was determined based on the average of unilateral bite force in the premolar region (210–420 N), being 70% inferior to the molar region [14]. The occlusal force was simulated in this research as an axial force on the middle of the pontic. However, clinically, occlusal forces are also present on the abutment teeth within a normal range between 210 and 420 N. Therefore, the forces were normalized to 300 N, without taking into consideration different directions of occlusal forces [15].

Structured calculation consists of establishing a system of equations for the movement of all nodes of the mesh, and, following their resolution, the approximation of the fields of deformations and stresses was deduced [16]. This method leads to the calculation of the field of movement in each node and the components of deformations and stresses in each element. These quantities are the solutions of the differential equation system. The values of the FEA are presented as maximum and minimum main constraints [16].

Von Mises stress distribution and maximum strain were evaluated separately at the cement layer and on the zirconium dioxide bridge. Von Mises stress is a theoretical stress value that represents the comparison between the general 3D stress and the uni-axial stress yield limit (Simscale), which is the maximum voltage stress limit that a specific material can retain without losing energy or experiencing permanent deformation. Thus, it was necessary to evaluate the equivalent elastic stress and deformation of von Mises, as well as the shear stress and maximum elastic deformation. Elastic deformation (initial deformation/length) is the maximum possible deformation without permanent deformation caused by the 3D combination of stresses in the case of von Mises stress or maximum shear deformation by shear stress. Both constraints are the result of the applied nodal force mentioned above.

## 3. Results

Table 3 and Table 4 present the von Mises stress and the total deformation distribution on the inlay bridge and in the cement layer. The stress distribution (Table 3) was mainly localized among the bridge on the connector area of the pontic in all the evaluated study groups. The stress distribution among the bridge was higher for 0.4 mm thickness, specifically for the cement with lower elastic modulus. The highest stress value was observed for the Variolink 0.4 group, while the lowest was noted for the Panavia 0.2 group. Concerning the cement layer, the highest stress value was observed with the Variolink 0.4 group, while the lowest was obtained with the Panavia 0.2 group. The highest total deformation value (Table 4) was found for the Variolink 0.2 group, while the lowest was observed for the Panavia 0.4 group.

The FEA values of maximum stress and deformation are presented in Table 5.

## 4. Discussion

In the present study, the influence of elastic moduli and thickness of the resin cement on the stress distribution and the maximum strain generated in RBFPD was investigated by FEA. It was observed that a resin cement with a higher elastic modulus and in a thickness of 0.2 mm generated a more favorable stress distribution within the zirconium-dioxide-based inlay bridge. The correlation between the elastic modulus and the thickness of the cement and the mechanical performance of the inlay bridge was observed. According to this finding, both null hypotheses tested were rejected.

Based on a comparison using a ratio of stress distribution/maximum deformation, Panavia can generate slightly less stress than Variolink for the same strain. These findings are supported by the literature [5,8] and Hooke’s law [17]. It was stated that adhesive cement with low elastic modulus reduces the tensile stress concentration in the cement layer for an inlay bridge. However, it increased the stress concentration in the bridge connector. Hence, it can be stated that the more elastic the cement resin is, the higher stress it can generate to the zirconium dioxide inlay bridge. Consequently, when a rigid restorative material is used, a higher risk of failure in the adhesive interface layer is expected [5,18]. This adhesive interface includes three different structures: the intaglio surface of the inlay bridge, resin cement, and the cavity surface. Each structure exhibited different elastic modulus (dentin—18.6 GPa; enamel—84.1 GPa; Panavia F2.0—12 GPa; Variolink II Ivoclar—8.3 GPa; zirconium dioxide—200 GPa), resulting in different stress concentrations [8]. A similar study that used zirconium dioxide found a higher stress concentration for Variolink (25.56 MPa) when compared to Panavia cement (14.78 MPa) for 0.07 mm thickness, at the adhesive interface of both abutment teeth [18].

It is worth emphasizing that a more favorable stress distribution along the adhesive interface for the cement thickness of 0.2 mm was observed. The optimal result was found for Panavia with a thickness of 0.2 mm. When compared to the 0.4 mm models (Panavia and Variolink), the 0.2 mm cement was able to generate less stress distribution even with a slightly smaller strain. The present results are supported by another study [19], denoting that a thin resin-based cement layer is favorable. It is worth mentioning that the decrease in tensile bond for the cement thickness exceeding 35 μm was noted [20]. However, it was stated that different thicknesses of the cement resin did not influence the mechanical performance of occlusal veneers cemented on molars [21]. Thus, these phenomena can be explained with a different restoration design and, therefore, stress distribution. Furthermore, the axial force (600 N) exerted on the occlusal veneer in a previous study was significantly higher compared to the inlay bridge used in this FEA study (300 N).

Panavia and Variolink resin cements were selected for the present study because they are often used in FEA and clinical study. Additionally, cements with extreme values of elastic modulus were applied to better illustrate the influence of mechanical properties on the stress distribution [22,23,24,25,26,27].

The RBFPDs used in this study were made of zirconium dioxide. This material exhibits a high fracture resistance (1247 N), while the fracture resistance for lithium disilicate amounts up to 1000 N [28]. Moreover, materials with a lower elastic modulus such as lithium disilicate (82.3 ± 18.3 GPa) result in a lower stress distribution in the bridge [29]. Moreover, there was no difference between a posterior inlay monolithic zirconium-dioxide-based and a chromium cobalt-based bridge, porcelain layered under a load of 400 N in the FEA study. Interestingly, a slight advantage (however insignificant) regarding stress bearing for zirconia, when compared to the chromium cobalt substructure and porcelain coating, was noted [30].

It is worth mentioning that untreated zirconium dioxide exhibits a low bond strength (17.02 MPa) to resin cement compared to lithium disilicate (28.7 MPa). Therefore, premature de-bonding is more often observed in these cases [31,32]. Remarkably, after application of the primer on the zirconium dioxide surface, the bond strength to cement amounts up to 21.248 ± 8.971 MPa [33].

The connectors used in this study have a circular shape with an area of 16 mm^2^. Moreover, the connector area was the most prone to damage; hence, no fracture was noted. This finding is supported by another study [34]. This type of connector was chosen based on previous literature, suggesting that the minimum connector cross-section area amounts up to 12–16 mm^2^ for the posterior restorations [35].

Despite the complex simulations that FEA can create, this mathematical analysis generates theoretical results that need to be correlated with laboratory and clinical findings. This correlation allows a full understanding of the failure mode of the analyzed structures and seems to better imitate clinical scenarios than in vitro studies [36].

Caution must be exercised when interpreting these results. Temperature changes, pH, humidity, and polymerization shrinkage (PS) were not simulated in this analysis. The cement layer was homogeneous without defects or bubbles. Furthermore, aging of the bridge and the resin cement was not studied. Next, zirconium dioxide primer and bonding layer were not included in this research, because the elastic modulus and Poisson’s ratio of the primer and the bonding are not available in the literature. Thus, additional tests were needed to calculate these parameters. Moreover, these values seem to be negligible and would not influence the final results.

In this research, only axial loading was studied without taking into account articulation and mastication forces. Nevertheless, in clinical conditions, occlusal loads are also distributed on the abutment teeth. However, in this research, loadings were only exercised on the pontic tooth [37]. Additionally, occlusal and parafunctional forces may differ (300 to 1200 N) among patients depending on age, sex, tooth position, and temporomandibular disorders. Yet, in the present study, the occlusal force was set to 300 N and localized on the center of the occlusal surface of the pontic. Both abutment teeth can move independently in all directions. Hence, further studies should analyze the relationship between the PS of different thicknesses using different cement resin on the bond strength of the bridge. Nonetheless, clinical studies should be performed to verify the obtained results. Furthermore, it is important to mention that one outcome variable is not sufficient to elucidate material behavior. For example, the resins are viscoelastic therefore other materials properties such as creep should also be considered. Similarly, since the oral conditions are dynamic, fatigue and dynamic mechanical analysis should be considered in future research.

## 5. Conclusions

Within the limitations of this study, 0.2 mm layer Panavia F2.0 seemed to exhibit a more favorable stress distribution when used for cementation of posterior zirconium dioxide-based RBFPD. However, both studied materials possessed clinically acceptable properties.

## Figures and Tables

**Table 1:** Elastic modulus and Poisson’s ratio of the different elements used in numerical simulations [5,12,13].

Materials	Elastic Modulus (GPa)	Poisson’s Ratio
Zirconium dioxide	200	0.31
Enamel	84.1	0.33
Dentin	18.6	0.32
Resin cement (Panavia F2.0)	12	0.33
Resin cement (Variolink II Ivoclar)	8.3	0.24
Periodontal ligament	0.069	0.45
Spongious bone	1.37	0.30
Cortical bone	13.7	0.30

**Table 2:** Study groups.

Study Group	Type of Cement	Cement Thickness
Panavia 0.2	Panavia F2.0 (Kuraray Dental, Tokyo, Japan)	0.2 mm
Panavia 0.4		0.4 mm
Variolink 0.2	Variolink II (Ivoclar Vivadent Inc, Amherst, NY, USA)	0.2 mm
Variolink 0.4		0.4 mm

**Table 3:** Stress distribution on the inlay bridge and the cement layer for both Variolink and Panavia, 0.2 and 0.4 mm.

		Cement Thickness	
		0.2 mm	0.4 mm
Inlay bridge	Variolink	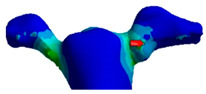	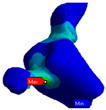
	Panavia	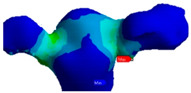	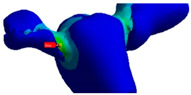
Cement layer	Variolink	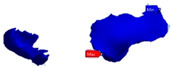	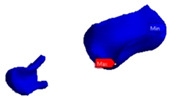
	Panavia	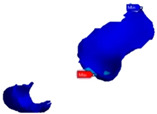	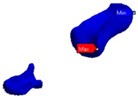

**Table 4:** Strain distribution on the inlay bridge and the cement layer for both Variolink and Panavia, 0.2 and 0.4 mm.

		Cement Thickness	
		0.2 mm	0.4 mm
Inlay bridge	Variolink	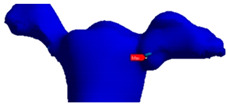	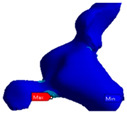
	Panavia	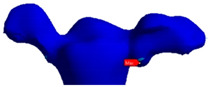	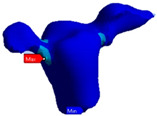
Cement layer	Variolink	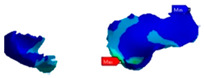	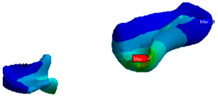
	Panavia	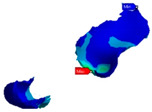	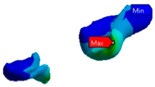

**Table 5:** Maximum stress and maximum deformation in cement layer.

Study Groups	0.2 mm		0.4 mm	
	Stress Distribution (Pa)	Maximum Deformation (m)	Stress Distribution (Pa)	Maximum Deformation (m)
Panavia	1.07 × 10^6^	2.95 × 10^−4^	1.45 × 10^6^	2.33 × 10^−4^
Variolink	1.12 × 10^6^	3.36 × 10^−4^	1.76 × 10^6^	2.84 × 10^−4^

## Data Availability

Not applicable.
